# *Faecalibacterium prausnitzii* increases following fecal microbiota transplantation in recurrent *Clostridioides difficile* infection

**DOI:** 10.1371/journal.pone.0249861

**Published:** 2021-04-09

**Authors:** Olle Björkqvist, Ignacio Rangel, Lena Serrander, Cecilia Magnusson, Jonas Halfvarson, Torbjörn Norén, Malin Bergman-Jungeström

**Affiliations:** 1 Department of Gastroenterology, Faculty of Medicine and Health, Örebro University, Örebro, Sweden; 2 School of Medical Sciences, Örebro University, Örebro, Sweden; 3 Division of Clinical Microbiology, Department of Clinical and Experimental Medicine, Linköping University, Linköping, Sweden; 4 Department of Biomedical and Clinical Sciences, Linköping University, Linköping, Sweden; 5 Department of Infectious Diseases, Region Jönköping County, Jönköping, Sweden; 6 Faculty of Medicine and Health, Department of Laboratory Medicine, National Reference Laboratory for *Clostridioides Difficile*, Clinical Microbiology, Örebro University, Örebro, Sweden; INRAE, FRANCE

## Abstract

**Objective:**

Fecal microbiota transplantation (FMT) is a highly effective treatment for *Clostridioides difficile* infection (CDI). However, the fecal transplant’s causal components translating into clearance of the CDI are yet to be identified. The commensal bacteria *Faecalibacterium prausnitzii* may be of great interest in this context, since it is one of the most common species of the healthy gut microbiota and produces metabolites with anti-inflammatory properties. Although there is mounting evidence that *F*. *prausnitzii* is an important regulator of intestinal homeostasis, data about its role in CDI and FMT are relatively scarce.

**Methods:**

Stool samples from patients with recurrent CDI were collected to investigate the relative abundance of *F*. *prausnitzii* before and after FMT. Twenty-one patients provided fecal samples before the FMT procedure, at 2 weeks post-FMT, and at 2–4 months post-FMT. The relative abundance of *F*. *prausnitzii* was determined using quantitative polymerase chain reaction.

**Results:**

The abundance of *F*. *prausnitzii* was elevated in samples (N = 9) from donors compared to pre-FMT samples (N = 15) from patients (adjusted P<0.001). No significant difference in the abundance of *F*. *prausnitzii* between responders (N = 11) and non-responders (N = 4) was found before FMT (P = 0.85). In patients with CDI, the abundance of *F*. *prausnitzii* significantly increased in the 2 weeks post-FMT samples (N = 14) compared to the pre-FMT samples (N = 15, adjusted P<0.001). The increase persisted 2–4 months post-FMT (N = 15) compared to pre-FMT samples (N = 15) (adjusted P<0.001).

**Conclusions:**

FMT increases the relative abundance of *F*. *prausnitzii* in patients with recurrent CDI, and this microbial shift remains several months later. The baseline abundance of *F*. *prausnitzii* in donors or recipients was not associated with future treatment response, although a true predictive capacity cannot be excluded because of the limited sample size. Further studies are needed to discern whether *F*. *prausnitzii* plays an active role in the resolution of CDI.

## Introduction

The annual incidence of *Clostridioides difficile* infection (CDI) in Sweden is 65/100,000 as compared with 147/100,000 in the United States, translating into nearly half a million cases and 29,000 deaths [1, 2]. Roughly 20% of patients experience recurrent disease after a period of an initial response to CDI treatment [2, 3]. Recurrent CDI is associated with an increased mortality risk compared to the index episode and most patients do not experience sustained cure after antibiotics [4, 5].

The onset of the CDI is most often preceded by a course of antibiotic treatment that translates into structural and functional disruptions of the normal host microbiome [6]. Consequently, colonization resistance is lost, allowing *C*. *difficile* spores to germinate into vegetative cells, resulting in clinical infection. Because dysbiosis of the gut microbiota plays a pivotal role in the pathogenesis of CDI [7], it is intuitive to treat the infection with bacteriotherapy. Three randomized controlled studies have demonstrated that fecal microbiota transplantation (FMT) is a highly effective treatment for recurrent CDI [8–10].

Metagenomic research has shown that FMT successfully reverses the dysbiosis [11], and after FMT, the microbiota of treated patients resembles that of the donor [12]. Differences between donors in terms of gut microbiota composition could in part explain the reported variation in post-FMT cure rates of 70–95% [13–15]. However, the fecal transplant’s causal components translating into clearance of the CDI are yet to be identified. Identifying such mediators would be most helpful given that the information could be used to predict treatment response and identify suitable donors.

A reduction of the commensal bacteria *Faecalibacterium prausnitzii* has been reported in other diseases characterized by gut dysbiosis, including Crohn’s disease [16], ulcerative colitis [17], celiac disease [18], obesity [19], diabetes [20], and psoriasis [21]. *F*. *prausnitzii* is one of the most common species of the healthy gut microbiota, representing >5% of the total bacterial count [22]. The bacteria produce at least two metabolites with anti-inflammatory properties, the short-chain fatty acid butyrate and the protein microbial anti-inflammatory molecule [23]. Although there is mounting evidence that *F*. *prausnitzii* is an important regulator of intestinal homeostasis, data about its role in CDI and FMT are relatively scarce. To examine the dynamics of *F*. *prausnitzii* in patients receiving FMT due to recurrent CDI, we conducted a longitudinal study to quantify the abundance of *F*. *prausnitzii* before and after treatment. We hypothesized that *F*. *prausnitzii* is depleted in patients with recurrent CDI and that the baseline abundance of the bacteria in donors or recipients may be used to predict treatment response.

## Material and methods

### Study design

In this dual-center study stool samples from patients with recurrent CDI were collected to investigate the relative abundance of *F*. *prausnitzii* before and after FMT. The patients were asked to provide fecal samples within 2 days before the FMT procedure, at 2 weeks (±7 days) post-FMT, and finally, 2–4 months (±5 days) post-FMT. Fecal samples were collected locally at each study site and stored at -80°C until DNA extraction. The relative abundance of *F*. *prausnitzii* was determined using a quantitative polymerase chain reaction (qPCR) approach, targeting the 16S rRNA gene.

Two criteria were used to define treatment response: (1) resolution of diarrhea within 14 days of the FMT and (2) absence of symptoms associated with relapse during the follow-up of 2–4 months post-FMT. Patients had to meet both criteria to be classified as responders; otherwise, they were classified as non-responders.

### Study population

We included consecutive patients treated with FMT for recurrent CDI at the Department of Infectious Diseases, Linköping University Hospital, Linköping (n = 15) and Ryhov Hospital, Jönköping (n = 6) between November 2015 and November 2017. All participants submitted written consent before inclusion. Patients who provided at least 2/3 of the requested fecal samples were included. In addition to this inclusion criterion, patients with underlying colonic comorbidity (colonic cancer, N = 1 and lymphocytic colitis, N = 1) were excluded, since it would have been difficult to evaluate the clinical response of the FMT in these patients.

Recurrent CDI was defined as a relapse of clinical symptoms combined with a positive laboratory test for *C*. *difficile* within 8 weeks of the previous episode of CDI. This definition is in accordance with the clinical guidelines from the European Society of Clinical Microbiology and Infectious Diseases [24]. Patients were treated based on local clinical guidelines and referred for FMT at the second relapse of CDI after failing previous treatment with metronidazole, vancomycin or fidaxomicin.

Medical records were reviewed by an experienced infectious disease specialist at each hospital to confirm the diagnosis of CDI and record information on symptoms and antibiotic use during the study.

Healthy, unrelated donors were recruited from the general population. All donors were screened through questionnaires, serologic testing and fecal culturing to avoid the transference of pathogenic microorganisms. The fecal transplants were kept in -80° C until the day of transplantation. The transplant was mixed with 0,25 liter of NaCl solution and delivered as a rectal enema. Patients were advised to stay in supine position for 30 minutes after administration. The pairing of donors and patients was determined by transplant availability only and no matching according to age or sex was applied. The study was approved by the Regional Ethics Committee in Linköping (DNR 2014/484-31).

### DNA extraction

DNA was extracted from 100 mg of feces using QIAamp PowerFecal DNA kit (Qiagen, Hilden, Germany) according to the manufacturer’s instructions. The extraction started with 5 minutes bead beating at 30Hz in TissueLyser II (Qiagen) and further extraction was performed in QIAcube (Qiagen) automation. The DNA quantification was executed in a Qubit 2.0 fluorometer (Life Technologies) according to Qubit dsDNA high sensitivity, HS, assay (Thermo Fisher Scientific). After quantification, the DNA samples were diluted to 5 ng/μL in Ultrapure water.

### qPCR

Each PCR reaction was performed with 20 ng of template DNA and measured in triplicates. The HOT FIREPol^®^ EvaGreen^®^ qPCR was used to detect PCR products on an Applied Biosystems 7900HT Fast Real-Time PCR System (Life Technologies). All samples were measured in triplicates. The ΔΔ-method was used to calculate the abundance of *F*. *prausnitzii* in relation to the total bacterial count [25], and the total bacterial abundance was measured using eubacterial primers. Thermal cycle conditions [26], primer sequences for *F*. *prausnitzii* (Willing B. et al.) and Eubacteria (Barman et al.) are described elsewhere [27, 28].

### Statistics

The abundance of *F*. *prausnitzii* was expressed as the log-2 of the fold change related to the total bacterial count. To exclude the potential effect of anti-CDI antibiotics, analyses of follow-up samples were restricted to patients who had not been treated with metronidazole, vancomycin or fidaxomicin after FMT. Some patients in this study had incomplete data (e.g., only 2/3 requested fecal samples were provided). Because of this collection limitation, the overall difference in abundance of *F*. *prausnitzii* before and after FMT was primarily assessed using the Mann-Whitney U test (unpaired analysis). To account for the dependence between samples, the nonparametric Wilcoxon matched-pairs signed-rank test was used for the comparisions of samples that had been provided at two adjacent timepoints, e.g. before and after FMT (paired analysis). To adjust for multiple comparison between groups in the longitudinal analysis, the Benjamini-Hochberg method was applied with a false discovery rate of 0.05. Raw P values were adjusted for all six comparisons between groups. Pearson’s correlation coefficient was used to evaluate the relation of the abundance of *F*. *prausnitzii* between donors and recipients. The Benjamini-Hochberg adjusted P values were computed in R-studio (version 1.3.1093) using the function “p.adjust”. All other statistical calculations were performed in GraphPad Prism, version 8.

## Results

### Clinical cohort

**Fig 1:**
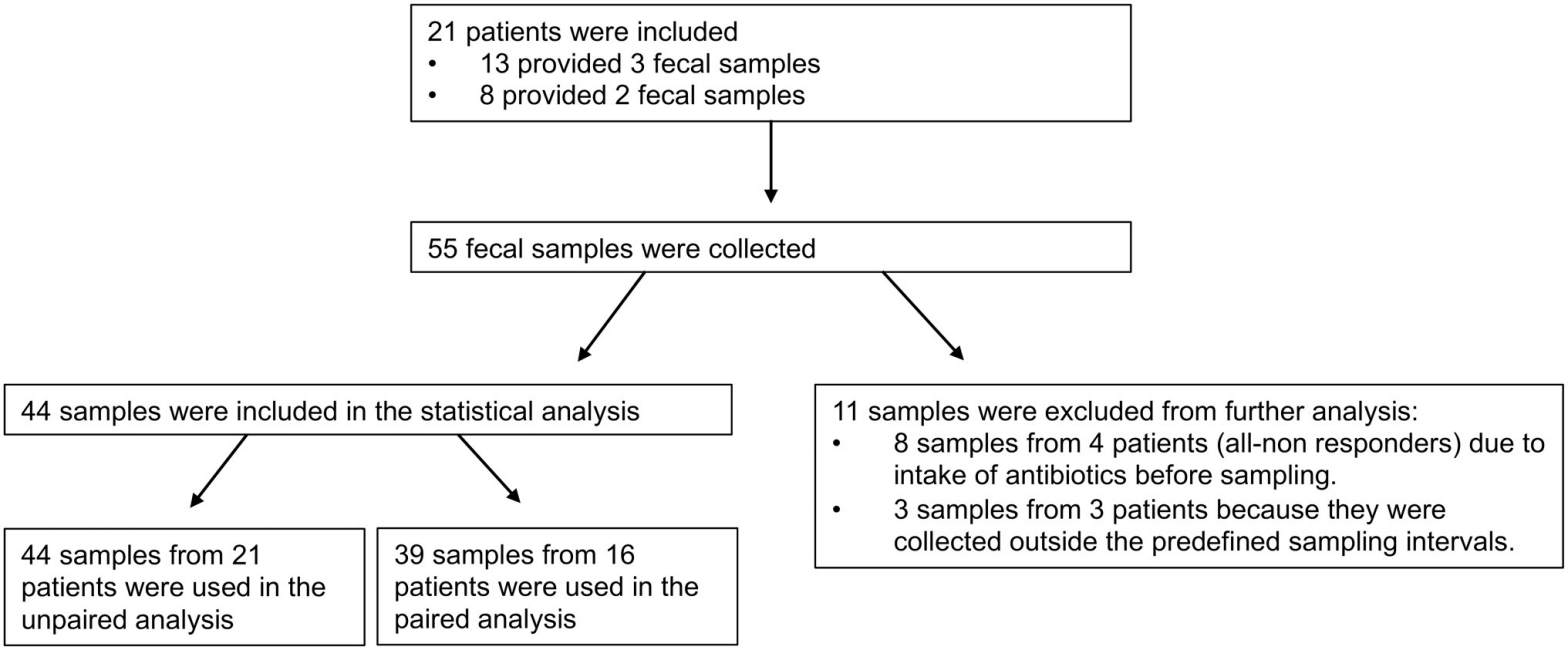
Flow chart of included patients and samples used in the statistical analysis.

In total, 21 patients met the inclusion criteria and formed the study population. Eleven patients provided only 2/3 of requested fecal samples, resulting in 55 samples collected during the study period. Eight unrelated donors contributed with 21 transplants to the study participants. Basic demographics and clinical characteristics of the study population are presented in Table 1. Of the 21 patients, 17 responded to the FMT treatment and four were non-responders. The four non-responders received CDI antibiotics (Vancomycin, N = 3; Fidaxomicin, N = 1) after the FMT and before the sampling 2 weeks later. Accordingly, the fecal samples (N = 8) from these four patients, collected after antibiotic prescription, were excluded from the analysis. Moreover, three samples from three patients were collected outside the predefined sampling intervals (7–21 days post-FMT and 55–125 days post-FMT) and therefore excluded from analysis (see Fig 1).

**Table 1:** Basic demographics and clinical characteristics.

**Total patients, N**	21
**Females, N (%)**	11 (52.3)
**Age in years, median (IQR)**	76 (9)
**Number of recurrences before FMT, median (IQR)**	2 (1)
**Treatment for the last recurrence before FMT**	
Vancomycin, N (%)	21 (100)
**Response to FMT**	
Responder, N	17
Non-responder, N	4

IQR, interquartile range; FMT, fecal microbiota transplantation.

### No association between abundance of *F*. *prausnitzii* and treatment response in patients and donors before FMT

**Fig 2:**
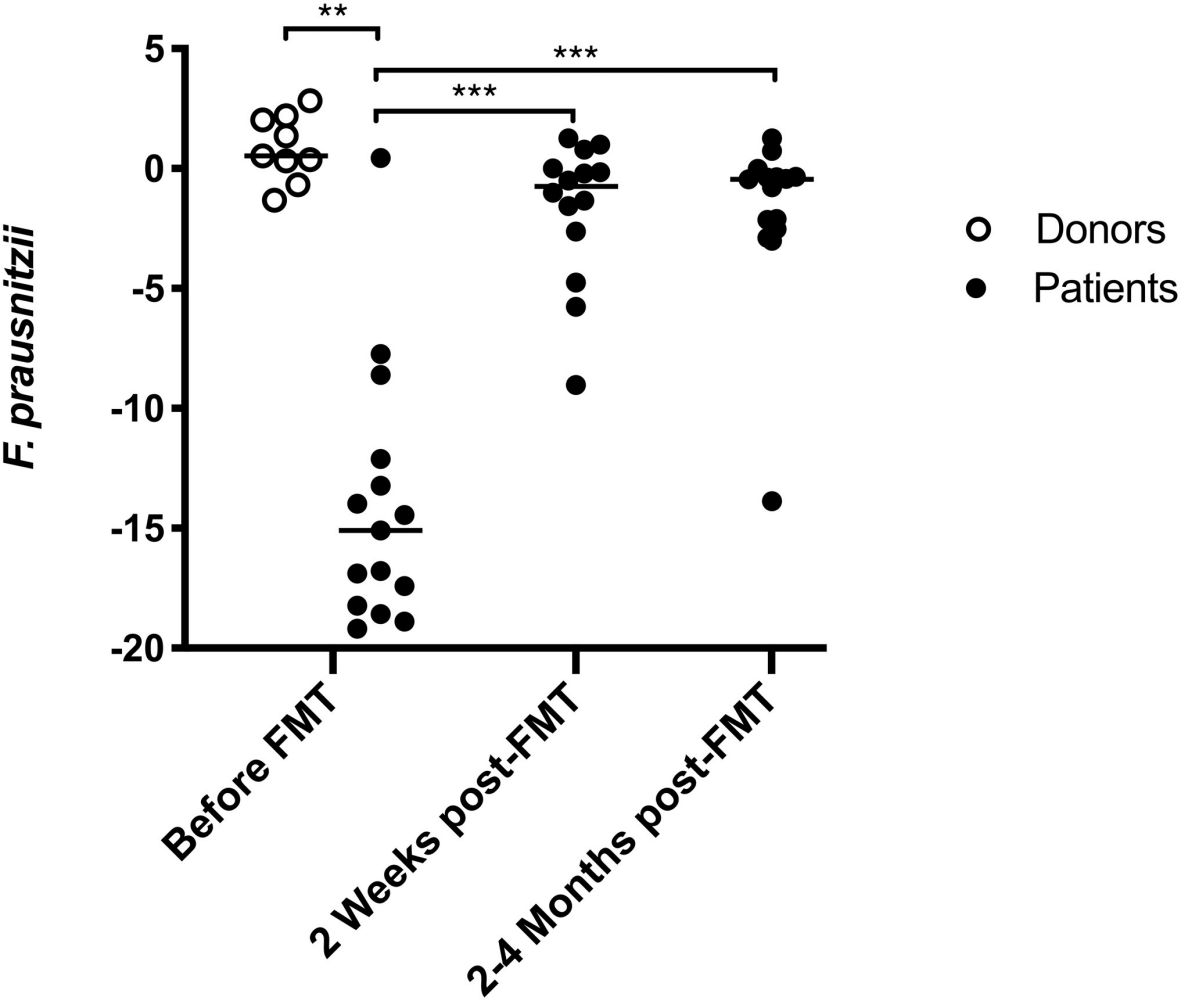
Abundance of *F*. *prausnitzii* before and after fecal microbiota transplantation (FMT) in 21 patients with *C*. *difficile* infection (CDI). The abundance of *F*. *prausnitzii* was elevated in donors compared with patients’ pre-FMT samples. A significant increase in the abundance of *F*. *prausnitzii* in patients’ samples was observed 2 weeks post-FMT, which was sustained at 2–4 months after FMT. Y-axis: The abundance of *F*. *prausnitzii* was expressed as the log-2 of the fold change related to the total bacterial count. The Mann-Whitney U test was used for comparisons between groups. Highly significant differences in the median abundance between groups are marked with *** (adjusted P < 0.001) or ** (adjusted P < 0.01). The horizontal lines indicate the group median.

The abundance of *F*. *prausnitzii* was elevated in donors (N = 9) compared to patients’ pre-FMT samples (N = 15) (difference between medians = 15.6, adjusted P < 0.001 (Fig 2). No significant difference in the abundance of *F*. *prausnitzii* between responders (N = 11) and non-responders (N = 4) was found before FMT (difference between medians = −3.2, P = 0.85), but the comparison was compromised by the limited number of samples. The abundance of *F*. *prausnitzii* in transplants provided to responders (N = 7) and non-responders (N = 2) appeared similar (difference between medians = −0.31), but the groups were too small to allow any statistically meaningful comparison.

### The abundance of *F*. *prausnitzii* increases after FMT

Using all fecal samples in the unpaired analysis, the abundance of *F*. *prausnitzii* significantly increased in the patients’ samples (N = 14) 2 weeks post-FMT compared to pre-FMT samples (N = 15) (difference between medians = 14.3, adjusted P < 0.001) (Fig 2). The increase persisted 2–4 months post-FMT (N = 15) compared to pre-FMT samples (N = 15) (difference between medians = 14.6, adjusted P < 0.001) (Fig 2). Based on the paired analysis, a similar increase in the abundance of *F*. *prausnitzii* was observed 2 weeks (adjusted P = 0.016) and 2–4 months (adjusted P = 0.012) post-FMT (Table 2).

**Table 2:** Results of the Wilcoxon matched-pairs signed-rank test.

Comparison	Number of pairs	Median of differences	P value	Adjusted p value
2 weeks post-FMT vs pre-FMT	9	15.9	0.012	0.016
2–4 months post-FMT vs pre-FMT	9	16.4	0.004	0.012
2–4 months post-FMT vs 2 weeks post-FMT	12	−0.49	0.57	0.57

FMT, fecal microbiota transplantation.

Although the abundance of *F*. *prausnitzii* increased substantially following FMT, some minor, but statistically significant, differences in the relative abundance of *F*. *prausnitzii* remained between patients and donors, both at 2 weeks (differences between medians = −1.3, adjusted P = 0.016) and 2–4 months post-FMT (differences between medians = −1.0; adjusted P = 0.010). In a linear correlation analysis of donor-recipient pairs, the abundance of *F*. *prausnitzii* in an individual donor did not correlate with the abundance in the recipient’s sample two weeks post-FMT (R^2^ = 0.05, P = 0.52).

## Discussion

This study shows an increased relative abundance of *F*. *prausnitzii* after FMT in patients with recurrent CDI. The abundance of *F*. *prausnitzii* was notably higher in donors than in recipients at baseline (i.e. pre-treatment). Already 2 weeks post-FMT, the levels of *F*. *prausnitzii* approached those of the donors and this shift remained at 2–4 months post-FMT. Although the observed increase in *F*. *prausnitzii* after FMT indicates that the bacteria could be important in resolving CDI, the baseline abundance of *F*. *prausnitzii* in recipients and donors was not predictive of future treatment response.

While the microbiota dynamics have been extensively studied in CDI patients receiving fecal transplantation, only a few previous study have reported a significant increase in *F*. *prausnitzii* after FMT [29, 30]. Other studies may have failed to detect *F*. *prausnitzii* as most of these studies used next-generation sequencing (NGS) techniques. Although NGS provides detailed information on gut microbiota composition, the method sometimes fails to provide comprehensive data at the species level due to insufficient sequencing depth. Of note, several studies have reported that CDI is characterized by a depletion of the family *Ruminococcaceae*, to which the species *F*. *prausnitzii* belongs [31, 32]. In the current study we used qPCR to measure the relative abundance of *F*. *prausnitzii*. This method may result in a more specific and reliable quantification of a single species compared to massively parallel sequencing techniques, including NGS.

Our data on the increase of *F*. *prausnitzii* after FMT is supported by some previous studies [29, 30, 33, 34]. Intriguingly, Mintz et al. also used qPCR to measure *F*. *prausnitzii* and observed an increased level after FMT in a cohort of 14 patients with recurrent CDI [29]. This finding was recently replicated in a study of 26 patients [30], who performed an in-depth characterization of the gut microbiota composition using an untargeted metagenomic sequencing approach. Additionally, expansion of *F*. *prausnitzii* post-FMT have also been reported in a case report, and in a study of nine pediatric patients with CDI, although the observed increase was not statistically significant in these studies [33, 34].

It is not yet clear whether FMT’s effect is mediated by a few key species or by complex interactions between the donors and recipients’ entire gut microbiome. An experimental mouse study indicates that a transfer of only six phylogenetically diverse species may be sufficient to trigger a shift in the gut environment that facilitates re-expansion of the recipient’s commensal gut microbiota [35]. In 2013, Petrof et al. used a bacterial cocktail of 33 commensal species, including *F*. *prausnitzii*, as a successful treatment for two patients with antibiotic resistant CDI [36]. Selective bacteriotherapy has then been evaluated in a case series of 55 patients, and although conceptually successful, the remission rate reached only 64% [37], considerably lower compared to most FMT studies [15]. These findings indicates that although a few key species seem sufficient to inhibit *C*. *difficile* germination, transfer of an unfiltered microbiota with a higher diversity may provide a more robust and effective way to eradicate recurrent CDI.

Because this study is descriptive, it does not provide evidence that *F*. *prausnitzii* plays a causal role in resolving CDI. However, a previous study has shown that *C*. *difficile* induces inflammation by activating the nuclear factor κB (NF-κB) pathway [38]. In contrast, experimental studies have showed that *F*. *prausnitzii* is a major producer of short-chain fatty acids (e.g. butyrate), which in turn inhibits signaling through the (NF-κB) pathway [16, 39]. *F*. *prausnitzii* does also contribute to gut homeostasis by modulating the intestinal mucus barrier [40]. Moreover, increased levels of SCFA in feces have been reported after successful FMT [41].

Our study has several limitations. An important issue is whether the abundance of *F*. *prausnitzii* differs between responders and non-responders after FMT. *In vitro* studies suggest that *F*. *prausnitzii* is susceptible to vancomycin [42]. Because all four non-responders in our study were treated with CDI-antibiotics before a post-FMT fecal sample was secured, this issue could not be addressed. Ideally, future studies should collect fecal samples both before and after antibiotic exposure to determine the importance of dysbiosis in non-responders after FMT. Another limitation is that the qPCR-based approach in our study quantifies both viable and non-viable bacteria. Research shows that oxygen exposure during transplant preparation diminishes the proportion of viable *F*. *prausnitzii* [43]. Finally, eight patients provided only two of the three fecal samples sought. The missing data resulted in a decreased statistical power and reduced the possibility to identify potentially true alterations in the abundance of *F*. *prausnitzii*, including comparison of donors and recipients. However, the observed sizable increase in the abundance of *F*. *prausnitzii* after FMT is unlikely to be significantly altered due to missing data.

This paper demonstrates that *F*. *prausnitzii* increases after FMT, a finding that may be underreported in the literature owing to methodological limitations of sequencing studies. The baseline abundance of *F*. *prausnitzii* in donors or recipients was not associated with future treatment response, although a true predictive capacity cannot be excluded because of the limited sample size. Further studies are needed to discern whether *F*. *prausnitzii* plays an active role in the resolution of CDI.

## Supporting information

S1 Table Demographic data of donors. (PDF)

S1 File Source data for this study. (XLSX)

## References

[1] Rizzardi K, Norén T, Aspevall O, Mäkitalo B, Toepfer M, Johansson Å, et al. National Surveillance for Clostridioides difficile Infection, Sweden, 2009–2016. Emerg Infect Dis. 2018;24: 1617–1625. 10.3201/eid2409.171658 30124193 PMC6106436

[2] Lessa FC, Mu Y, Bamberg WM, Beldavs ZG, Dumyati GK, Dunn JR, et al. Burden of Clostridium difficile infection in the United States. N Engl J Med. 2015;372: 825–834. 10.1056/NEJMoa1408913 25714160 PMC10966662

[3] Eyre DW, Walker AS, Wyllie D, Dingle KE, Griffiths D, Finney J, et al. Predictors of First Recurrence of Clostridium difficile Infection: Implications for Initial Management. Clin Infect Dis. 2012;55: S77–S87. 10.1093/cid/cis356 22752869 PMC3388024

[4] Olsen MA, Yan Y, Reske KA, Zilberberg MD, Dubberke ER. Recurrent Clostridium difficile infection is associated with increased mortality. Clinical Microbiology and Infection. 2015;21: 164–170. 10.1016/j.cmi.2014.08.017 25658560

[5] McFarland LV, Elmer GW, Surawicz CM. Breaking the cycle: treatment strategies for 163 cases of recurrent Clostridium difficile disease. Am J Gastroenterol. 2002;97: 1769–1775. 10.1111/j.1572-0241.2002.05839.x 12135033

[6] Theriot CM, Koenigsknecht MJ, Carlson PE, Hatton GE, Nelson AM, Li B, et al. Antibiotic-induced shifts in the mouse gut microbiome and metabolome increase susceptibility to Clostridium difficile infection. Nat Commun. 2014;5: 3114. 10.1038/ncomms4114 24445449 PMC3950275

[7] Fuentes S, van Nood E, Tims S, Jong IH, ter Braak CJ, Keller JJ, et al. Reset of a critically disturbed microbial ecosystem: faecal transplant in recurrent *Clostridium difficile* infection. The ISME Journal. 2014;8: 1621–1633. 10.1038/ismej.2014.13 24577353 PMC4817604

[8] van Nood E, Vrieze A, Nieuwdorp M, Fuentes S, Zoetendal EG, de Vos WM, et al. Duodenal infusion of donor feces for recurrent Clostridium difficile. N Engl J Med. 2013;368: 407–415. 10.1056/NEJMoa1205037 23323867

[9] Cammarota G, Masucci L, Ianiro G, Bibbò S, Dinoi G, Costamagna G, et al. Randomised clinical trial: faecal microbiota transplantation by colonoscopy vs. vancomycin for the treatment of recurrent Clostridium difficile infection. Alimentary Pharmacology & Therapeutics. 41: 835–843. 10.1111/apt.13144 25728808

[10] Hvas CL, Dahl Jørgensen SM, Jørgensen SP, Storgaard M, Lemming L, Hansen MM, et al. Fecal Microbiota Transplantation Is Superior to Fidaxomicin for Treatment of Recurrent Clostridium difficile Infection. Gastroenterology. 2019;156: 1324–1332.e3. 10.1053/j.gastro.2018.12.019 30610862

[11] Kellingray L, Gall GL, Defernez M, Beales ILP, Franslem-Elumogo N, Narbad A. Microbial taxonomic and metabolic alterations during faecal microbiota transplantation to treat Clostridium difficile infection. Journal of Infection. 2018;77: 107–118. 10.1016/j.jinf.2018.04.012 29746938

[12] Khoruts A, Dicksved J, Jansson JK, Sadowsky MJ. Changes in the composition of the human fecal microbiome after bacteriotherapy for recurrent Clostridium difficile-associated diarrhea. J Clin Gastroenterol. 2010;44: 354–360. 10.1097/MCG.0b013e3181c87e02 20048681

[13] Barnes D, Ng K, Smits S, Sonnenburg J, Kassam Z, Park KT. Competitively Selected Donor Fecal Microbiota Transplantation: Butyrate Concentration and Diversity as Measures of Donor Quality. Journal of Pediatric Gastroenterology and Nutrition. 2018;67: 185–187. 10.1097/MPG.0000000000001940 29470297

[14] Brandt L, Aroniadis O, Mellow M, Kanatzar A, Kelly C, Park T, et al. Long-Term Follow-Up of Colonoscopic Fecal Microbiota Transplant for RecurrentClostridium difficileInfection. American Journal of Gastroenterology. 2012;107: 1079–1087. 10.1038/ajg.2012.60 22450732

[15] Quraishi MN, Widlak M, Bhala N, Moore D, Price M, Sharma N, et al. Systematic review with meta-analysis: the efficacy of faecal microbiota transplantation for the treatment of recurrent and refractory Clostridium difficile infection. Alimentary Pharmacology & Therapeutics. 46: 479–493. 10.1111/apt.14201 28707337

[16] Sokol H, Pigneur B, Watterlot L, Lakhdari O, Bermúdez-Humarán LG, Gratadoux J-J, et al. Faecalibacterium prausnitzii is an anti-inflammatory commensal bacterium identified by gut microbiota analysis of Crohn disease patients. Proc Natl Acad Sci U S A. 2008;105: 16731–16736. 10.1073/pnas.0804812105 18936492 PMC2575488

[17] Machiels K, Joossens M, Sabino J, De Preter V, Arijs I, Eeckhaut V, et al. A decrease of the butyrate-producing species Roseburia hominis and Faecalibacterium prausnitzii defines dysbiosis in patients with ulcerative colitis. Gut. 2014;63: 1275–1283. 10.1136/gutjnl-2013-304833 24021287

[18] Palma GD, Nadal I, Medina M, Donat E, Ribes-Koninckx C, Calabuig M, et al. Intestinal dysbiosis and reduced immunoglobulin-coated bacteria associated with coeliac disease in children. BMC Microbiol. 2010;10: 1–7. 20181275 10.1186/1471-2180-10-63 PMC2843610

[19] Furet J-P, Kong L-C, Tap J, Poitou C, Basdevant A, Bouillot J-L, et al. Differential Adaptation of Human Gut Microbiota to Bariatric Surgery–Induced Weight Loss. Diabetes. 2010;59: 3049–3057. 10.2337/db10-0253 20876719 PMC2992765

[20] Navab-Moghadam F, Sedighi M, Khamseh ME, Alaei-Shahmiri F, Talebi M, Razavi S, et al. The association of type II diabetes with gut microbiota composition. Microbial Pathogenesis. 2017;110: 630–636. 10.1016/j.micpath.2017.07.034 28739439

[21] Eppinga H, Sperna Weiland CJ, Thio HB, van der Woude CJ, Nijsten TEC, Peppelenbosch MP, et al. Similar Depletion of Protective Faecalibacterium prausnitzii in Psoriasis and Inflammatory Bowel Disease, but not in Hidradenitis Suppurativa. J Crohns Colitis. 2016;10: 1067–1075. 10.1093/ecco-jcc/jjw070 26971052

[22] Petra Louis, Flint Harry J. Diversity, metabolism and microbial ecology of butyrate‐producing bacteria from the human large intestine. FEMS Microbiology Letters. 2009;294: 1–8. 10.1111/j.1574-6968.2009.01514.x 19222573

[23] Quévrain E, Maubert MA, Michon C, Chain F, Marquant R, Tailhades J, et al. Identification of an anti-inflammatory protein from Faecalibacterium prausnitzii, a commensal bacterium deficient in Crohn’s disease. Gut. 2016;65: 415–425. 10.1136/gutjnl-2014-307649 26045134 PMC5136800

[24] Debast SB, Bauer MP, Kuijper EJ. European Society of Clinical Microbiology and Infectious Diseases: Update of the Treatment Guidance Document for Clostridium difficile Infection. Clinical Microbiology and Infection. 2014;20: 1–26. 10.1111/1469-0691.12418 24118601

[25] Livak KJ, Schmittgen TD. Analysis of Relative Gene Expression Data Using Real-Time Quantitative PCR and the 2−ΔΔCT Method. Methods. 2001;25: 402–408. 10.1006/meth.2001.1262 11846609

[26] Björkqvist O, Repsilber D, Seifert M, Brislawn C, Jansson J, Engstrand L, et al. Alterations in the relative abundance of Faecalibacterium prausnitzii correlate with changes in fecal calprotectin in patients with ileal Crohn’s disease: a longitudinal study. Scand J Gastroenterol. 2019;54: 577–585. 10.1080/00365521.2019.1599417 31104514

[27] Willing B, Halfvarson J, Dicksved J, Rosenquist M, Järnerot G, Engstrand L, et al. Twin Studies Reveal Specific Imbalances in the Mucosaassociated Microbiota of Patients with Ileal Crohn’s Disease. Inflamm Bowel Dis. 2009;15: 653–660. 10.1002/ibd.20783 19023901

[28] Barman M, Unold D, Shifley K, Amir E, Hung K, Bos N, et al. Enteric Salmonellosis Disrupts the Microbial Ecology of the Murine Gastrointestinal Tract. Infect Immun. 2008;76: 907–915. 10.1128/IAI.01432-07 18160481 PMC2258829

[29] Mintz M, Khair S, Grewal S, LaComb JF, Park J, Channer B, et al. Longitudinal microbiome analysis of single donor fecal microbiota transplantation in patients with recurrent Clostridium difficile infection and/or ulcerative colitis. PLOS ONE. 2018;13: e0190997. 10.1371/journal.pone.0190997 29385143 PMC5791968

[30] Mullish BH, McDonald JAK, Pechlivanis A, Allegretti JR, Kao D, Barker GF, et al. Microbial bile salt hydrolases mediate the efficacy of faecal microbiota transplant in the treatment of recurrent Clostridioides difficile infection. Gut. 2019;68: 1791–1800. 10.1136/gutjnl-2018-317842 30816855 PMC6839797

[31] Song Y, Garg S, Girotra M, Maddox C, von Rosenvinge EC, Dutta A, et al. Microbiota Dynamics in Patients Treated with Fecal Microbiota Transplantation for Recurrent Clostridium difficile Infection. PLOS ONE. 2013;8: e81330. 10.1371/journal.pone.0081330 24303043 PMC3841263

[32] Antharam VC, Li EC, Ishmael A, Sharma A, Mai V, Rand KH, et al. Intestinal Dysbiosis and Depletion of Butyrogenic Bacteria in Clostridium difficile Infection and Nosocomial Diarrhea. J Clin Microbiol. 2013;51: 2884–2892. 10.1128/JCM.00845-13 23804381 PMC3754663

[33] Moelling K, Broecker F. Fecal microbiota transplantation to fight Clostridium difficile infections and other intestinal diseases. Bacteriophage. 2016;6: e1251380. 10.1080/21597081.2016.1251380 28090385 PMC5221744

[34] Hourigan SK, Ahn M, Gibson KM, Pérez-Losada M, Felix G, Weidner M, et al. Fecal Transplant in Children With Clostridioides difficile Gives Sustained Reduction in Antimicrobial Resistance and Potential Pathogen Burden. Open Forum Infect Dis. 2019;6. 10.1093/ofid/ofz379 31660343 PMC6790402

[35] Lawley TD, Clare S, Walker AW, Stares MD, Connor TR, Raisen C, et al. Targeted Restoration of the Intestinal Microbiota with a Simple, Defined Bacteriotherapy Resolves Relapsing Clostridium difficile Disease in Mice. PLOS Pathogens. 2012;8: e1002995. 10.1371/journal.ppat.1002995 23133377 PMC3486913

[36] Petrof EO, Gloor GB, Vanner SJ, Weese SJ, Carter D, Daigneault MC, et al. Stool substitute transplant therapy for the eradication of Clostridium difficile infection: ‘RePOOPulating’ the gut. Microbiome. 2013;1: 1–12. 24467987 10.1186/2049-2618-1-3 PMC3869191

[37] Tvede M, Tinggaard M, Helms M. Rectal bacteriotherapy for recurrent Clostridium difficile-associated diarrhoea: results from a case series of 55 patients in Denmark 2000–2012. Clin Microbiol Infect. 2015;21: 48–53. 10.1016/j.cmi.2014.07.003 25636927

[38] Jefferson KK, Smith MF, Bobak DA. Roles of Intracellular Calcium and NF-κB in the Clostridium difficile Toxin A-Induced Up-Regulation and Secretion of IL-8 from Human Monocytes. The Journal of Immunology. 1999;163: 5183–5191. 10553038

[39] Segain JP, Raingeard de la Blétière D, Bourreille A, Leray V, Gervois N, Rosales C, et al. Butyrate inhibits inflammatory responses through NFkappaB inhibition: implications for Crohn’s disease. Gut. 2000;47: 397–403. 10.1136/gut.47.3.397 10940278 PMC1728045

[40] Wrzosek L, Miquel S, Noordine M-L, Bouet S, Chevalier-Curt MJ, Robert V, et al. Bacteroides thetaiotaomicron and Faecalibacterium prausnitzii influence the production of mucus glycans and the development of goblet cells in the colonic epithelium of a gnotobiotic model rodent. BMC Biol. 2013;11: 61. 10.1186/1741-7007-11-61 23692866 PMC3673873

[41] Seekatz AM, Theriot CM, Rao K, Chang Y-M, Freeman AE, Kao JY, et al. Restoration of short chain fatty acid and bile acid metabolism following fecal microbiota transplantation in patients with recurrent Clostridium difficile infection. Anaerobe. 2018;53: 64–73. 10.1016/j.anaerobe.2018.04.001 29654837 PMC6185828

[42] Martín R, Miquel S, Benevides L, Bridonneau C, Robert V, Hudault S, et al. Functional Characterization of Novel Faecalibacterium prausnitzii Strains Isolated from Healthy Volunteers: A Step Forward in the Use of F. prausnitzii as a Next-Generation Probiotic. Frontiers in Microbiology. 2017;8: 1226. 10.3389/fmicb.2017.01226 28713353 PMC5492426

[43] Chu ND, Smith MB, Perrotta AR, Kassam Z, Alm EJ. Profiling Living Bacteria Informs Preparation of Fecal Microbiota Transplantations. PLOS ONE. 2017;12: e0170922. 10.1371/journal.pone.0170922 28125667 PMC5268452

