# Supplementary material for: Faecalibacterium prausnitzii increases following fecal microbiota transplantation in recurrent Clostridioides difficile infection
Source: PLoS One. 2021 Apr 9;16(4):e0249861. doi: 10.1371/journal.pone.0249861 (PMC8034738; doi:10.1371/journal.pone.0249861)
Supplement: S1 Table — (PDF) [file pone.0249861.s001.pdf]

S1 Table. Basic demographics of donors.

Study center	Age	Sex
Linköping	43	Male
Linköping	34	Male
Linköping	59	Female
Linköping	41	Female
Jönköping	61	Male
Jönköping	43	Male
Jönköping	49	Male
Jönköping	46	Female
